# Big Data and the Study of Social Inequalities in Health: Expectations and Issues

**DOI:** 10.3389/fpubh.2018.00312

**Published:** 2018-10-26

**Authors:** Cyrille Delpierre, Michelle Kelly-Irving

**Affiliations:** ^1^Inserm, UMR1027, Université Toulouse III, Toulouse, France; ^2^Institut Fédératif d'études et de Recherches Interdisciplinaires Santé Société (Iferiss), Toulouse, France

**Keywords:** global health, social determinants of health, embodiment, Big data, health policy, health equity

## Abstract

Understanding the construction of the social gradient in health is a major challenge in the field of social epidemiology, a branch of epidemiology that seeks to understand how society and its different forms of organization influence health at a population level. Attempting to answer these questions involves large datasets of varied heterogeneous data suggesting that Big Data approaches could be then particularly relevant to the study of social inequalities in health. Nevertheless, real challenges have to be addressed in order to make the best use of the development of Big Data in health for the benefit of all. The main purpose of this perspective is to discuss some of these challenges, in particular: (i) the perimeter and the particularity of Big Data in health, which must be broader than a vision centerd solely on care, the individual and his or her biological characteristics; (ii) the need for clarification regarding the notion of data, the validity of data and the question of causal inference for various actors involved in health, such data as researchers, health professionals and the civilian population; (iii) the need for regulation and control of data and their uses by public authorities for the common good and the fight against social inequalities in health. To face these issues, it seems essential to integrate different approaches into a close dialog, integrating methodological, societal, and ethical issues. This question cannot escape an interdisciplinary approach, including users or patients.

## Social gradient of health

The social gradient in health refers to the observation that, on average as one descends the social gradient toward increasing disadvantage, one's health worsens. This phenomenon has been widely observed with regard to social inequalities in mortality. For example, in a study examining the risk of cumulative mortality over time from age 40 to 65 in Europe, Gallo et al. noted that as education levels decrease, the risk of mortality increases in a graded and proportionate manner (1). The social gradient in health thus affects the entire population, without dichotomising the most advantaged vs. the most disadvantaged.

This social gradient in health is observed in most countries, including low, medium and high income countries, according to wealth, education or occupation. It covers a wide range of pathological processes that are not explained by traditional risk factors (such as health behaviors) alone. The social gradient in health occurs in men and women, possibly through different mechanisms, and it manifests itself very early in life, particularly through a developmental health gradient. Clyde Hertzman (2) described the social gradient in health as a “social fact” referring to Durkheim's definition (3).

## Biological embedding and the life course approach

Understanding the construction of the social gradient in health is a major challenge in the field of social epidemiology, a branch of epidemiology that seeks to understand how society and its different forms of organization influence health at a population level. Life course epidemiology provides a relevant conceptual framework by approaching health as the result of multiple combined exposures (chemical, physical, behavioral, psychosocial) socially differentiated and likely to modify biological processes that may promote the long-term development of pathologies (4). The way in which these different exposures express themselves at the biological level refers to the concept of embodiment. This concept has been developed in epidemiology by Nancy Krieger (5) and by Clyde Hertzman in studies of human development under the term “biological embedding” (6).

Research using a life course framework to study embodiment processes has shown that social inequalities in health are built up from an early stage. A seminal work often used in social epidemiology to illustrate this early construction of social inequalities in health is called the “Barker's hypothesis.” In the early 1990s, Barker et al. observed a relationship between intrauterine growth retardation and an increased risk of cardiovascular and metabolic diseases in adulthood (7), introducing the concept of Developmental Origins of Health and Disease (DOHaD). Since then, a large body of research has shown associations between a variety of exposures in early life and different health outcomes. Fundamental questions remain: through which mechanisms or pathways does the social environment become biological? How does the social environment alter normal biological functioning to promote the development of pathologies over time? Attempting to answer these questions involves large datasets of varied heterogeneous data. Life course epidemiology is largely based on longitudinal data, particularly birth cohorts, in which social, behavioral, environmental, and biological data are collected prospectively. Such databases nevertheless remain expensive and rare. Given these issues the contribution of Big Data could be particularly relevant. Notably, France, like other countries in Europe with a national health care system, has “large” representative databases, i.e., data coming from tax or health care systems or administrative data from reimbursement of health care consumption that may be used to study social inequalities in health. However, the use of big data to study health requires prior consideration that health has specificities that raise particular issues and problems that must be taken into account.

## Beyond a basic need, health as a special need

Health presents the characteristic of conditioning opportunities to participate in society, of being an essential component of human capital and an essential dimension of the human ability to pursue life's goals and ambitions. This particularity of health is well reflected in the WHO Constitution of 1946 (8) that states that health is one of the fundamental rights of every human being and that health of all peoples is a fundamental condition for world peace and security and that the ability to achieve the highest attainable health status is a fundamental right of every human being. The WHO definition of health as a “state of complete physical, mental and social well-being, not merely the absence of disease or infirmity” reflects this holistic view of health. The issue of health as a common good is therefore central to addressing the societal issues associated with the use of Big Data in health.

## Big data and the study social inequalities in health: high potential but underused in practice

Big Data could contribute to highlighting, documenting and analyzing the role of social determinants of health. This may be especially true through its potential to match social, occupational and environmental data with data of a clinical, biological, behavioral nature as well as health services data in original and innovative ways. This merging of data sources from medical systems, health insurance providers, clinical/ mortality registries, and hospitals with administrative and socio-demographic datasets is happening across countries, and may breath a new lease of life into epidemiological research. This may be especially true for the emerging use of social media in the landscape of health care. For one, it will allow for an increasingly wide variety of health determinants to be analyzed, and will facilitate exploratory analyses to identify new ones. These methodological developments could potentially generate knowledge from across institutional sectors taking interdisciplinary perspectives, using a wide diversity of variables.

However, this potentially broadened approach to health research that may be facilitated by Big Data is largely underused in practice. In health research, Big Data analyses currently remain focused on the use of large volumes of data, mainly biological in nature with a propensity for molecular and genome-level data, for individual purposes. This type of analysis is oriented toward determining individual risk or diagnostic decision-making through the analysis of vast amounts of individual biological data. The aim is to generate an “à la carte” health care, popularized by the term “personalized medicine” which is commonly used to refer to genomic medicine (9). Big datasets used for such analyses rarely contain information on the social context and environment (10). Consequently this usage of “Big Data” maintains that health is largely about disease and biological reductionism (11). To move health research beyond such an outdated and limited perspective the environmental, socioeconomic, psychological, and biological determinants of health, which make health research a complex, interdisciplinary and trans-dimensional field, need to be taken into account. Hypothesis generation on the multiple determinants of health involves merging a wide variety of data sources, including social media, from a diversity of databases. This means grappling with problems like different data structures, missing data, data validity, varied measurement and collection methods, including natural language and different disciplinary traditions. Herein lies both the huge challenge and vast potential of Big Data in contributing to population health research (11).

## Big data in health: some key scientific issues

### How valid are the data?

At a time when health decision making could be guided by data and algorithms, and more largely where some argue for a Big Data-driven science in which “the data speaks for itself” (12), it is becoming crucial to question Big Data validity for health research. One of the reason a lot of weight is attributed to Big Data analyses resides in flawed assumptions about massive datasets being quasi-complete, as opposed to being a sample from a population. But the extent to which the data being used are representative of a population is just as important a question in Big Data approaches as in any other quantitative field. The question arises in practice about population coverage and its representativeness with regard to the technology/tool used to generate data, with all the potential limitations that such work carried out on selected populations could have in terms of public health. This issue is particularly important when analyzing data from social media usage. Indeed such tools may not be used by everyone, and may exclude some parts of the population that may be particularly important to consider for studying social inequalities in health, or not used in the same manner which can have an impact on their completeness. Sociological studies that followed the implementation of informatics tools for health professionals showed that entire sections of the information needed for care continued to circulate in written form (13). Such “dark data” (12) will not be available in an analysis labeled as Big Data showing that the advent of Big Data approaches does not solve the problem of data representativeness and completeness, bias, measurement error. A “validity first” approach (14) is therefore more essential than ever with the developing use of Big data in health.

A concomitant risk could be to consider data as a-theoretical and a-political and to be treated as objective, neutral or even pure. However, using Big Data methods to carry out health research raises a number of questions as to the purpose for the data was originally collected and the objective behind their secondary uses to carry out research. The objective, method and quality that guide data collection shape the information that is produced. The way information is organized is the result of technical, commercial, political choices. Behind the production of data lies economic, commercial, and political issues. Data can be useful or relevant in a specific field or for a specific question, but not appropriate in another: one can then speak of the territory of validity of data. The theories, frameworks and inherent subjectivity of researchers, companies, public institutions or society as a whole continue to be paramount driver as to the nature of what data are collected and how they are assembled. Technical debates have the effect of depoliticizing these very real issues but far from suppressing any theory, modeling or a priori choices, Big Data analysis carries within it these notions.

### Is the association causal?

Causal inference is a central question in quantitative health research, and must remain so when applying Big Data techniques. Big data approaches are largely used to identify correlations for doing prediction, and not for an etiological purpose. Getting to grips with the causal structure of data may not be a useful or profitable exercise for businesses using Big Data, however in the field of health and health intervention this is essential. Intervening on factors to improve one health condition without first knowing if these factors are determinants of the health condition studied and without trying to understand the causal relationships between the various determinants involved is likely to be a pointless exercise. A concomitant major challenge involves understanding and controlling the “black-box” algorithms that are used to perform many analyses and thus to produce data and results (15). The increasing difficulty in understanding the algorithms used and their assumptions is creating a risk of loss of control for scientist and more broadly for people. Understanding what is behind these types of algorithms, their underlying assumptions, how they work, developing their access and transparency are key questions. Data users and researchers should question who has developed the algorithms, and to what end. Far from the end of theory (16), the search for causality and the meaning behind the data are still and always will be major issues, even more so in the specific field of health.

## Big data in health: some key societal issues

### How to use big data in health research?

The potential new usage of Big Data in health research requires us to think about the way health is apprehended. As such, the usage of Big Data challenges the public authority about the direction given or pursued regarding the management of health, prevention and the organization of the care system. This political vision of health is all the more crucial as many health systems are faced with two decisive and ambivalent turning points: the simultaneous personalization and commercialization of health. In many countries the healthcare model has been based on curative care relying on a singular relationship between general physician and patient. This individual view of health is reflected through the use of Big Data to develop “personalized” medicine that produces standards and norms that underlie individual responsibility regarding behaviors. This questions the compatibility of a system based on the health risk-sharing with the development of personalized medicine, based on an increasingly detailed analysis of individual risks via algorithms. Two visions of the individual can be schematically opposed: an “economic” vision of the individual as autonomous, rational and fully conscious of his choices and behaviors; and a “social” vision of the individual as a self-regulating being but with limited capacity for self-regulation, whose choices and behaviors are not necessarily subject to systematic individual and autonomous decision-making, but are an integral part of socially defined norms and interactions. The usage of Big Data can either be used to promote an economic vision of the individual (as logically private companies do), or a social vision of the individual. The principles of solidarity and universal access on which many health care systems in Western Europe are based may be challenged by the advent of Big Data. The balance between the common vs. the individual interest in the way health and health care system are structured is exacerbated by the differential potential uses of Big Data. Faced with a growing financial deficit in many universal health systems, one temptation may be to develop conditional access to the health care system based on the adoption of behaviors in accordance with recommendations from public authorities, or even private companies, with the risk that health and its standards reflect their own vision. In practice, this would lead to reinforcing institutional control over individuals, or introducing penalties in the case of non-compliance with prescribed treatments or behaviors, based on monitoring tools and the analysis of each individual's data. Such alterations and evolutions in health care systems may be incompatible with inclusive approaches taking into account the cultural and socioeconomic situations and capabilities of all users.

The ability of Big Data to reduce social inequalities in health will depend upon the ways in which public institutions will develop, use and promote available data. Past experiences regarding the diffusion and uptake of technological innovations across society suggest that attention needs to be paid to their impact on social inequalities in health and notably on populations whose access to these innovations is reduced or who are excluded from data collection. Research points to the value of an approach based on the concept of proportionate universalism for reducing social inequalities in health (17). This consists of promoting policies the intensity or amount of which is distributed proportionally to the needs of populations. The opportunities and/or limitations that Big Data in health research could represent for deploying such policies deserve to be considered.

### Which control and regulation?

Merging different databases naturally raises the issue of data confidentiality, privacy and the respect for people's rights. These rights can no longer be guaranteed in the era of database interoperability, which can technically make previously anonymous data identifiable. This is even truer with social media data where we can interrogate what privacy means in this field? The consequences on individual liberties and privacy, commercial or discriminatory practices are not merely marginal side effects. This danger is already widely apparent in various sectors of the economy, such as the finance or insurance sector, where companies use algorithms to adapt and tailor their products to our individual situations, without our knowledge (18). The question of data control is thus one major issue. Who controls the data? According to which business models? While in a big data approach data are used in ways that by definition are not anticipated a priori, and that all types of data can become “personal” after matching, how to ensure control of personal data for citizens? What is the future of free and informed consent when secondary and tertiary uses of the data cannot be foreseen? How can we reduce the asymmetry of power and information between companies and organizations that hold data on individuals, by empowering individuals not only to control the use of their data by others, but also to develop their own uses of their personal data? How to standardize data usage rules across countries and between private and public companies?

The evolution of the legal framework on data regulation and data sharing at the national level and international level will largely depend our future ability to take full advantage of the potential offered by Big Data for health research. This is a real challenge both to protect people's rights and at the same time to offer a sufficient and simple access to public data and not to favor the emergence of a competing offer from private databases that are potentially less sensitive to the protection of individuals' rights. These issues raise the question of the public control of the development of Big Data in health. The form of this regulation remains to be defined as do its missions, but they could include anticipating relevant uses (“prospective reflection”), defining needs to encourage the emergence of ideas, best practice guidelines (for research in particular). The modus operandi of this regulation will undoubtedly have to distinguish uses for the general interest from uses for the individual interest, as distinguishing private/commercial use from public/academic use.

## Conclusion

The main interest of big data in health is above all based on the cross-linking of data that are usually designed to be partitioned, produced in very diverse and numerous units of time and place, and the analyses of these data, not limited to the classical techniques used in biomedicine. As a result, Big Data in health generates very high expectations and hopes for a better understanding of health and better care for the benefit of all. Nevertheless, certain obstacles persist and constitute real challenges in order to make the best use of the development of Big Data in health for the benefit of all: (i) the perimeter of Big Data in health, which must be broader than a vision centred solely on care, the individual and his or her biological characteristics; (ii) a lack of training and knowledge of the various actors, health professionals and the civilian population, in order to better understand the notion of data and algorithms, in particular their validity; (iii) a need for regulation and control of data and their uses by public authorities for the common good and the fight against social inequalities in health. The societal issues related to Big Data in health are major questions that the public authorities must address in order to make the most of the contribution of Big Data. It seems essential to integrate the different approaches into a close dialogue, integrating methodological, societal and ethical issues. This question cannot escape an interdisciplinary approach, including users or patients.

## Author contributions

All authors listed have made a substantial, direct and intellectual contribution to the work, and approved it for publication.

### Conflict of interest statement

The authors declare that the research was conducted in the absence of any commercial or financial relationships that could be construed as a potential conflict of interest.
